# Bioengineering in salivary gland regeneration

**DOI:** 10.1186/s12929-022-00819-w

**Published:** 2022-06-06

**Authors:** Maryam Hajiabbas, Claudia D’Agostino, Julia Simińska-Stanny, Simon D. Tran, Amin Shavandi, Christine Delporte

**Affiliations:** 1 Laboratory of Pathophysiological and Nutritional Biochemistry, Faculty of Medicine, Université Libre de Bruxelles, 808 Route de Lennik, Blg G/E CP 611, B-1070 Brussels, Belgium; 2 Department of Process Engineering and Technology of Polymer and Carbon Materials, Faculty of Chemistry, Wroclaw University of Science and Technology, Norwida 4/6, 50-373 Wroclaw, Poland; 3 3BIO-BioMatter, École Polytechnique de Bruxelles, Université Libre de Bruxelles, Avenue F.D. Roosevelt, 50 - CP 165/61, 1050 Brussels, Belgium; 4 McGill Craniofacial Tissue Engineering and Stem Cells Laboratory, Faculty of Dental Medicine and Oral Health Sciences, McGill University, Montreal, QC H3A 0C7 Canada

**Keywords:** Salivary gland, Tissue engineering, Biomaterial, Cell culture models, Xerostomia

## Abstract

Salivary gland (SG) dysfunction impairs the life quality of many patients, such as patients with radiation therapy for head and neck cancer and patients with Sjögren’s syndrome. Multiple SG engineering strategies have been considered for SG regeneration, repair, or whole organ replacement. An in-depth understanding of the development and differentiation of epithelial stem and progenitor cells niche during SG branching morphogenesis and signaling pathways involved in cell–cell communication constitute a prerequisite to the development of suitable bioengineering solutions. This review summarizes the essential bioengineering features to be considered to fabricate an engineered functional SG model using various cell types, biomaterials, active agents, and matrix fabrication methods. Furthermore, recent innovative and promising approaches to engineering SG models are described. Finally, this review discusses the different challenges and future perspectives in SG bioengineering.

## Background

SG fulfills critical roles in oral health, and their dysfunction can result in extensive deterioration of oral function and other health manifestations [1]. The parotid, submandibular, sublingual, and numerous minor glands secrete saliva in response to a wide range of biochemical signals and environmental cues [2]. Saliva contains water, mucus, antibacterial compounds, electrolytes, and various enzymes, which perform various vital functions in digestion, speaking, chewing, swallowing, and maintaining teeth and gingival tissues [3]. Irreparable SG damage causes hyposalivation manifesting itself by dry mouth symptom (xerostomia) in patients suffering from an autoimmune disease such as Sjögren’s syndrome or treated by radiotherapy for head and neck cancers [4]. Sjögren's syndrome, mainly occurring in middle-aged and older women, affect between 400,000 and 3.1 million adults worldwide [5], and radiotherapy for head and neck cancer treatment is given annually to around 1 million new patients worldwide [6, 7]. Considering existing treatments for hyposalivation are palliative and temporarily alleviate xerostomia [8], re-engineering SG will offer permanent and effective solutions to restore salivation [9]. In 1999, Bruce Baum and colleagues indicated three significant ways to re-engineer salivary epithelial cell functions: redesigning secretory function, repairing hypofunctional SG, and developing artificial SG [10]. Since then, various tissue engineering strategies have been conducted to restore salivation, and some have led to clinical trials [11–19]. Indeed, clinical trials suggest stem cell therapy [16, 17, 19] combined with special cell culture methods such as spontaneous cell aggregation, hanging drop and rotating culture vessels [1, 20, 21] or scaffold material [22–25] to produce functional secretory epithelial organoids, and gene therapy [18] may offer new therapeutic options for radiation-induced xerostomia. Due to challenges related to keeping the functionality of SG cells, researchers have examined the potential benefit of using a combination of different biomaterials [26, 27] and cell types to provide the optimal implant material for SG tissue engineering applications [28–30]. Nevertheless, fabricating a fully formed functional SG replacement in a controlled manner remains a challenge [11]. This review aims to provide essential features to be considered for the bioengineering of an SG model. It provides an overview of the existing knowledge on SG anatomy, structure, and function; SG diseases and dysfunction; critical elements in the tissue engineering approach for SG regeneration; strategies and fabrication methods in SG bioengineering; as well as the current challenges and perspectives.

## Salivary gland anatomy, structure, and function

SG originates from epithelial branching morphogenesis [31], which is characterized by three major phases: firstly, the development of a relative undifferentiated branched structure involving acinar and ductal precursors together with the developing vasculature and nerves; secondly, the epithelial branching morphogenesis induced by neural crest-derived mesenchymal growth factors and other molecular important cues; and thirdly, the maturation process at which stage the glands are fully functional and well-differentiated [32, 33]. The innervation and vascularization of the SG advance in parallel with the formation and maturation of the glands [32, 33]. The intricate morphogenic and differentiation processes are controlled by multiple signaling networks of cell–cell [32]. Mesenchymal cells and epithelial layer provide the stromal ECM and basement membrane (BM). During the branching process, the ECM composition varies from region to region and directly regulates the SG maturation process [31].

**Fig. 1:**
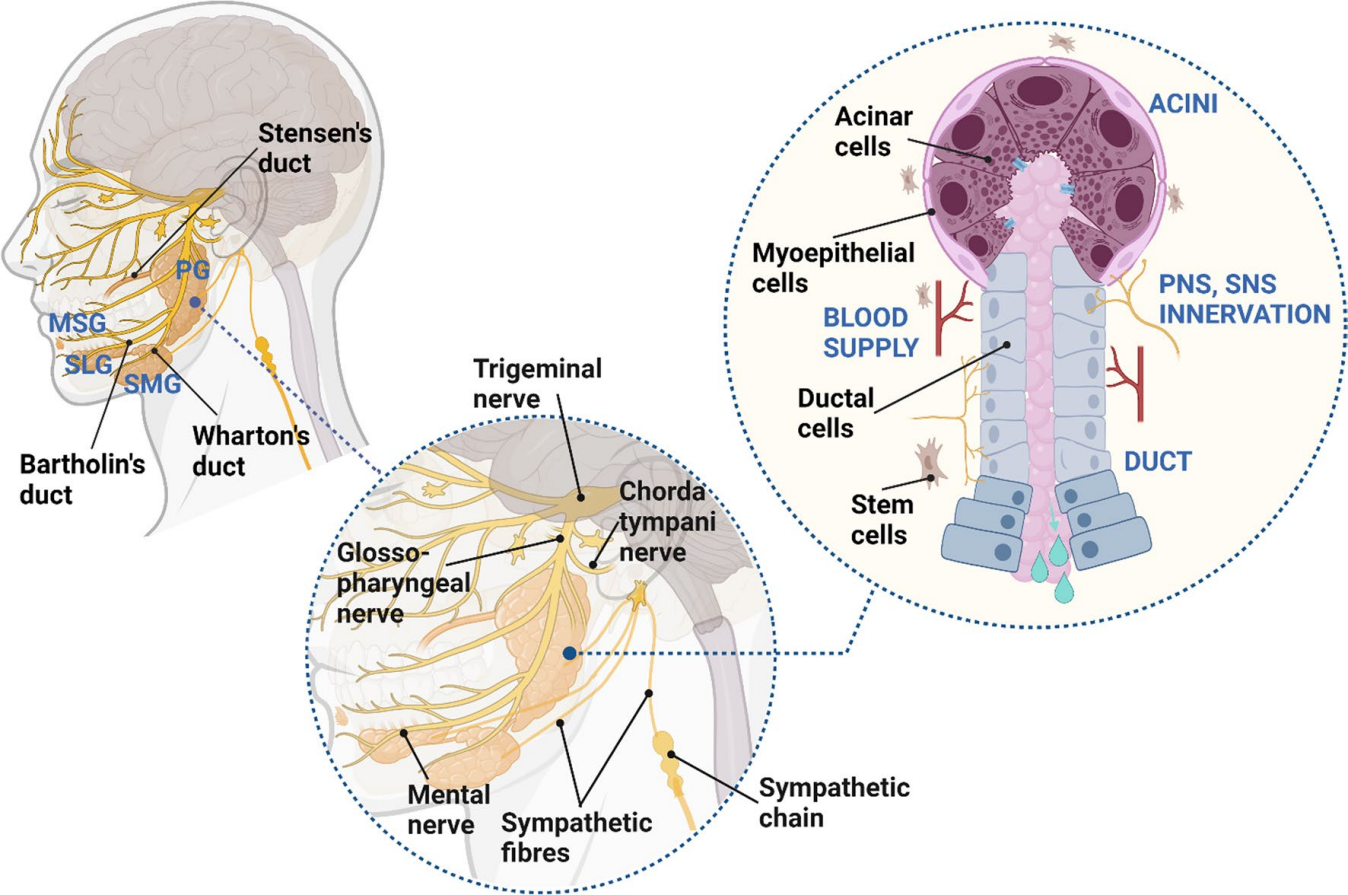
Human salivary gland features. **A** Schematic location of PG, SMG, SLG, and MSG in the oral cavity and the trigeminal nerve spreading postganglionic parasympathetic innervation structures of the head. **B** Schematic structure of one branch of human SGs, classification of different types of acini, innervation, blood supply (arteries), main excretory duct cells, and contribution of major and minor glands in resting or stimulating saliva flow

Human SG is made of three pairs of major glands known as parotid (PG), submandibular (SMG), and sublingual gland (SLG), collectively able to synthesize and secrete 90% of the total saliva, as well as comprising around 600–1000 minor glands (MSGs) distributed within the oral cavity that produce the remaining 10% of secreted saliva [34] (Fig. 1). Major glands share similar anatomical architecture: a branching duct with acini at one end and an opening to the oral cavity at the other end [33] (Fig. 1). The serous acini secrete a watery fluid rich in ions and proteins (such as a-amylase), while the mucous cells secrete a more viscous fluid rich in mucins [33]. The SMG and SLG are composed of a mixture of serous and mucous acini, unlike the PG exclusively made of serous acini [33]. Acini are surrounded by ECM, myoepithelial cells, myofibroblasts, immune cells, endothelial cells, stromal cells, and nerve fibers [33]. Myoepithelial cell contractility is regulated by the autonomic nervous system and supports the salivary flow by compressing the parenchymal structure and forcing fluid secretion into the ductal system [34].

**Fig. 2:**
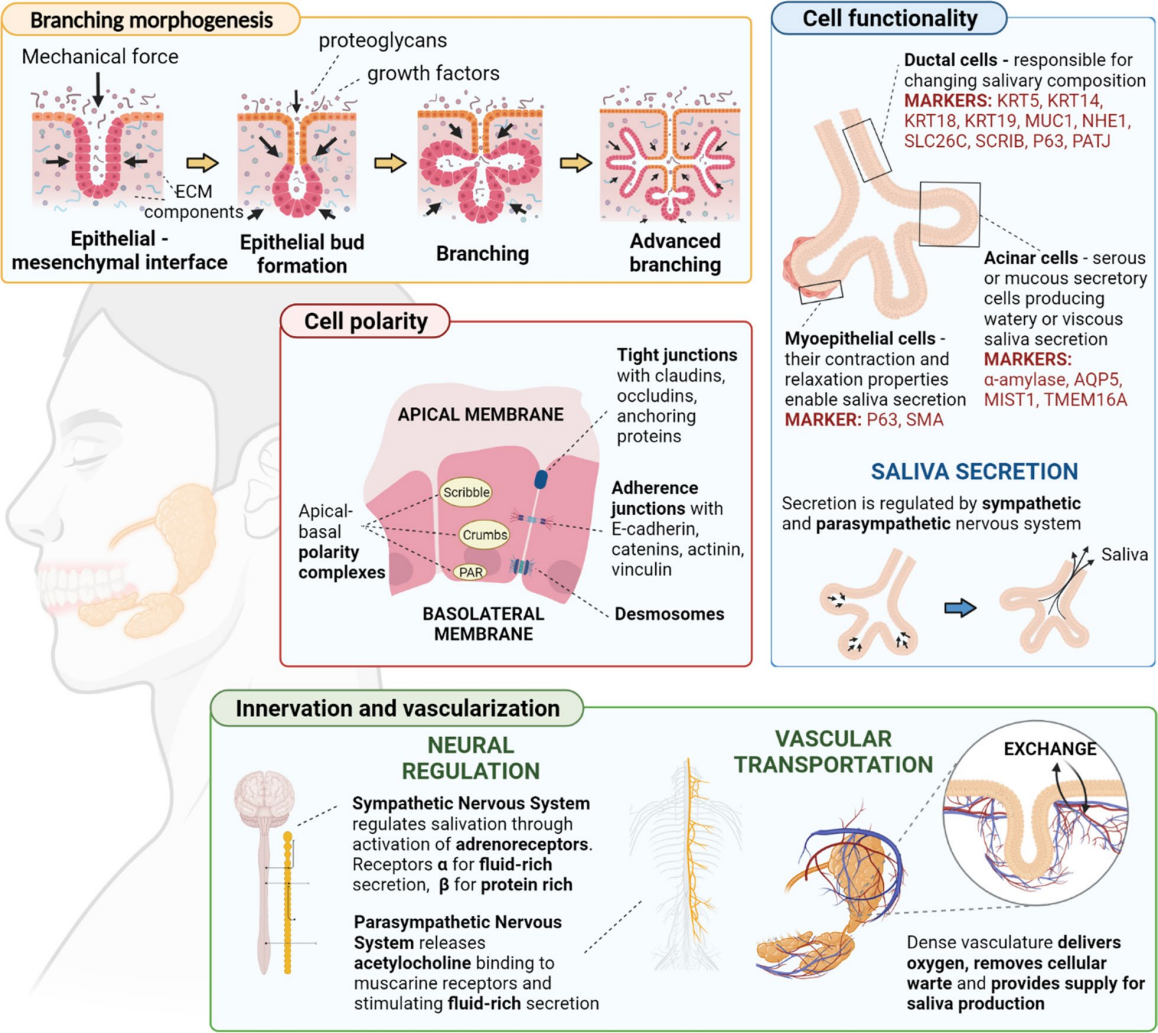
Schematic of key features for SG bioengineering (Figure drawn using Biorender.com)

Epithelial cell types can be identified using cell markers (Fig. 2). Partitioning-defective 1b (Par-1b) is involved in myoepithelial cell morphogenesis and differentiation during SG development and can be used as a myoepithelial cell marker in addition to SMA [35]. AQP5, a-amylase, AQP3, TMEM16A, and the transcription factor MIST1 are markers of acinar cells [11]. KRT5, KRT14, KRT18, KRT19, NHE1, and SLC26C are markers of ductal cells. Moreover, MUC1, along with the polarity markers Scribble Planar Cell Polarity Protein (SCRIB) and PATJ Crumbs Cell Polarity Complex Component, identify ductal cells in SG and hS/PCs progenitors [36]. Transcription factor P63 is expressed in myoepithelial cells and basal duct cells [32].

Once secreted by acinar cells, the primary saliva passes through an organized network of intercalated ducts, striated ducts, and main excretory ducts (Wharton’s duct for SMG; Stensen’s duct for PG; Bartolini duct for SLG; Rivinis ducts for MSG) [31, 33]. All major SGs ensure the production of daily saliva (0.5-1L per day) [37]. The capillaries diverging from the external carotid artery ensure the vascularizing of the PG, while the submental and sublingual arteries guarantee blood supply to the SMG and SLG [32]. The anatomical and structural features of SG are summarized in Fig. 1.

Saliva secretion is regulated by the interaction of different sensory signals activating afferent fibers of the facial (CNVII), glossopharyngeal (CNIX), and trigeminal (CNV) nerves [38]. The facial and glossopharyngeal nerves send the interneurons to the salivary centers [38]. The glossopharyngeal nerve synapses in the otic ganglion, and then the postganglionic parasympathetic fibers spread to the PG via the facial nerve synapsing in the submandibular ganglion and carrying on to the SLG and SMG [39] (Fig. 1). Sympathetic stimulation mainly results in acinar protein-rich secretion, while parasympathetic stimulation promotes the production of large volumes of saliva [38]. Acetylcholine binding to the muscarinic receptor (M3 receptors in PG and M3 and M1 in SMG) leads to inositol triphosphate and calcium signaling pathway activation and saliva secretion, while noradrenaline binding to beta1 adrenoreceptors leads to cyclic AMP signaling pathway [38]. In addition to these main neurotransmitters, some neuropeptides act as potential co-transmitters, such as substance P and VIP. Purinergic receptors are also involved in the control of SG secretion [40]**.** Crosstalk between the main neurotransmitter’s signaling pathways amplifies saliva flow and protein secretion under normal reflex conditions [34].

## Salivary gland diseases and dysfunction

SG dysfunction can lead to quantitative and/or qualitative changes in saliva composition and flow, leading to hyposalivation (manifesting as xerostomia) or hypersalivation (sialorrhea or hypersialia) [34, 41]. Xerostomia affects at least 10% of the adult population, with women and older people being more affected [42]. In opposition, sialorrhea, also known as drooling, may result from genuine SG alteration (primary sialorrhea), medication side effects, or may be associated with neurological disorders due to an impairment of voluntary oral motor activity or sensory ability (secondary sialorrhea) [34].

Due to its composition, saliva plays major role in food handling, teeth protection, and defense against microorganisms [43]. Therefore, persistent and severe SG hypofunction commonly predisposes patients to mucosal changes, caries, and other viral and bacterial infections [42]. The most common cause of SG hypofunction leading to xerostomia, especially in elderly people, is represented by the side effects of various medications [44, 45]. However, the adverse effects of drugs on salivary secretion are reversible. Numerous diseases and medical conditions can also affect SG function. SG inflammation, referred to as sialadenitis, can result from SG bacterial or viral infection or recurrent inflammatory disorders and is more prone to occur with hyposalivation and/or duct obstruction [46]. An incurable autoimmune disease, Sjögren’s syndrome, and radiation therapy for the treatment of head and neck cancer affect many individuals worldwide. Sjögren’s syndrome is a chronic autoimmune disorder and the second most prevalent rheumatic disease. It results from autoimmune epithelitis leading to partial destruction of exocrine glands due to a lymphoplasmacytic infiltration of the parenchyma [8, 47, 48]. The disease affects 0.1–4.8% of the population, with a female to male ratio of 9:1 [8]. The disease manifests as dry mouth (xerostomia) and dry eye (keratoconjunctivitis sicca) [8]. Currently, no efficient treatment exists for Sjögren’s syndrome, and only a number of tailor-made treatments are available to relieve the symptoms [8]. Radiation therapy is the most common or complementary lifesaving treatment for head and neck tumors, which can lead to irreversibly radiation-induced damage of SG [49]. Head and neck cancers usually begin in the squamous cells that cover the mucosal surfaces of the head and neck (the upper aerodigestive tract, paranasal sinuses, salivary, and thyroid gland) [49]. The most notable side effect of local irradiation is a reduction of saliva secretion due to an alteration of SG functionality. Recent study suggests the retained SG regenerative potential following radiation therapy may offer new avenues for therapeutic intervention [50].

The currently available treatments for xerostomia (stimulant medications or secretagogues, salivary substitutes, or artificial saliva) only provide temporary relief without offering a permanent solution [41]. Therefore, in search for potential and promising ways to permanently restore SG secretory function in patients with SG hypofunction, three main avenues have been considered: gene therapy [18, 51], stem cell therapies [16, 17, 19, 45, 52], and SG bioengineered models [9, 33, 53].

## Key features in bioengineering a SG model

A fully functional SG will require complex interactions among multiple cell types such as acinar, ductal, and myoepithelial cells in a branching structure, incorporating with the gland’s microenvironment that supplies it with vascular fluids and neural network. Consequently, an engineered SG must possess unique components and capabilities. An in-depth understanding of the mechanism regulating SG branching, SG cell polarity and secretion, innervation, and vascularization will provide valuable guidelines for engineering an SG model in vitro. Figure 2 shows the essential features that can be considered for proper functionality in SG bioengineered models.

### Branching morphogenesis

Branching morphogenesis is a critical process in SG formation [54]. The mechanical force generated by ECM, cell–cell interaction, and different growth factors in cell niche play essential roles in SG branching morphogenesis. These elements could provide structural guidance cues to help the gland keep its desired shape and organization.

Condensation or stretching of bundles of ECM fibrils by mesenchymal cells [55] and mesenchyme-generated traction forces [56] induced mechanical force capable of inducing branching morphogenesis. Although mechanical forces play a vital role in modulating branching structures, the underlying regulating mechanism is unclear. It remains necessary to deepen our understanding of the intracellular and extracellular forces involved in cell shape and morphogenesis [57]. Measuring the precise mechanical forces between cells and their microenvironment niche (ECM and neighbor cells) remains challenging. Biomechanics, mathematical modeling, and microfabricated techniques could provide an opportunity to evaluate cell mechanics in branching morphogenesis [58–60]. One of the next generations of advances in SG tissue engineering relies on the complete characterization of these forces to help design hydrogels with the appropriate stiffness and mechanical properties to control morphogenesis. Table 1 shows the ranges of some physical/mechanical parameters which can be applied to foster branching morphogenesis [60, 61].

**Table 1:** Expected ranges of some physical/mechanical parameters in branching morphogenesis

Parameter	Unit	Range
Epithelial viscosity	Kg m^−1^ s^−1^ = poise	10^4^–10^6^
Mesenchymal viscosity	Kg m^−1^ s^−1^ = poise	10^4^–10^6^
Epithelial surface tension	Kg s^−2^ = N/m	10^–3^–10^–2^
Clefting force	Kg m s^−2^ = N	10^–7^–10^–6^
Size of branching rudiment	m	10^–4^
The time scale of branching morphogenesis	s	10^4^–10^5^
The viscosity of embedding gel	Kg m^−1^ s^−1^ = poise	10^0^–10^6^
Cellular traction force	Kg m s^−2^ = N	10^–10^–10^–9^
ECM deformation length scale	m	10^–9^–10^–6^

Several ECM proteins accumulating in the clefs, such as collagen, laminin, and perlecan (located at the BM lining the mesenchymal-epithelial junction) are supposed to play a critical role in branching morphogenesis [55, 62]. Fibronectin and proteoglycans were shown to behave respectively as the putative cleft initiator [63] and inducer of branching morphogenesis through the release of fibroblast growth factor 10 (FGF10) [64]. The size and sulfation patterns of heparan sulfate present in proteoglycans modulate the biological activity of FGF10 and thereby epithelial branching morphogenesis [65]. Moreover, the ECM is extensively remodeled by matrix metalloproteinase (MMP) cleavage during branching morphogenesis [66]. Due to the vital role of the ECM in initiating and maintaining branching morphogenesis, numerous research studies have focused on designing hydrogels to grow salivary structures using material composition reproducing native ECM-cell interactions to support branching morphogenesis and cellular polarity [13, 63, 67–69], as well as appropriate chemical and physical cues promoting branching and reorganizing salivary structures with the proper orientation in 2D, 2.5D (monolayers on 3D substrates), and 3D-engineered models [15, 25, 69–73].

As mentioned above, changes in structure and cellular organization during branching morphogenesis could be guided by mechanical forces and dynamic interactions between neighboring cells. Epithelial (E)-cadherin plays a crucial role in cell–cell adhesion and increases significantly during SG development [54]. E-cadherin interacts via its C-terminus with various proteins such as β-catenin and forms homotypic interactions with other E-cadherin molecules on neighboring cells to mediate adhesive cell–cell interactions [74]. E-cadherin also plays an essential role in the cell self-organizing process since function-blocking anti-E-cadherin antibodies inhibit the process. Further, this protein is necessary for acinar differentiation and integrity of SG epithelial surfaces [75]. Despite constitutive epithelial E-cadherin expression throughout all stages of SG development, other critical cell–cell junctional proteins such as desmoplakins I/II and zonula occludens-1 (ZO-1) are rapidly lost [76]. It was therefore hypothesized that E-cadherin might act in place of other junctional proteins to stabilize external epithelial surfaces, which is significantly more robust between cell junctions of outer bud epithelial cells than those between the inner bud cells [63, 77]. Though, when a cleft forms to interrupt this layer of outer epithelial cells, E-cadherin localization and expression diminish as cell interactions shift from cell–cell to cell–matrix adhesion [63]. Although the exact role of E-cadherin in SG development remains vague at a mechanistic level, E-cadherin is crucially needed to regulate or mediate SG epithelial self-organization, branching, and acinus formation. Hence, analyzing E-cadherin expression in an SG artificial model can be helpful in evaluating cell–cell interaction for controlling morphogenesis.

Active agents are other cues controlling branching morphogenesis and thereby represent critical elements in tissue engineering approaches. Some growth factors are recognized as regulators for essential functions throughout all phases of embryonic development. The two most crucial growth factors for SG development are the epidermal growth factor (EGF) and the fibroblast growth factor (FGF). EGF promoted bud formation [78], while FGF7 and FGF10 differentially stimulated epithelial bud development and bud/duct elongation, respectively [79]. In addition, ex vivo studies reported that branching morphogenesis involves FGF receptor (FGFR) signaling [78, 80, 81] and EGF receptor (EGFR) signaling [82–84]. Other signaling pathways are also essential for SG branching morphogenesis, such as phospholipase Cγ1 (PLCγ1), mitogen-activated protein kinases (ERK-1/2), and phosphatidyl- inositol-3-kinase (PI3K) [79, 82, 85, 86]. These three signaling pathways employ multiple common downstream effectors during SG development that are crossregulated by other growth factor receptor systems. It was suggested that ERK-1/2 (stimulated by EGF and FGF7) is essential for bud formation, while PLCγ1 (stimulated by FGF7 and FGF10) may be important for bud/duct elongation [54]. Upstream regulators of FGFR signaling include the platelet-derived growth factor (PDGF) receptor signaling pathway that leads to the up-regulated FGF expression involved in SG branching morphogenesis [87]. These findings indicated the capability of growth factors to regulate complex arrays of interconnected signaling pathways to control morphogenesis. However, it is essential to continue evaluating how these factors and other different receptor-mediated signal transduction pathways communicate and how the ECM and cell–cell interactions control these signaling pathways and lead to the formation of a functional gland. Physical interactions between growth factors and ECM molecules also need to be considered in establishing morphogen gradients guiding SG branching morphogenesis [88, 89]. As such varying levels of growth factors matrix components might play a basic and unpredictable role in instructively patterning of various cellular functions, including differentiation, growth, and cell death. Moreover, osmotic gradient via ECM swelling and aquaporin water transport activity is another physical features that can induce dome formation to control the branching morphogenesis [89]. Understanding how morphogen gradients are created within ECM and their interplay with cellular and tissue level functions is one of the key gap knowledge limiting advancements in SG bioengineering.

### SG cell polarity

SG consists of tightly packed, polarized secretory acinar and absorptive ductal cells surrounded by myoepithelial cells, which are presumed to have contractile forces to ensure the proper unidirectional secretion of saliva. Therefore, obtaining polarized cells with accurate apical-basolateral positioning is essential for engineering a functional SG producing saliva. Epithelial cell polarity depends on different cues such as extracellular signals via BM components, cell–cell binding of asymmetrically distributed cadherins, and tight junctional components on lateral membranes. BM aids in cell sorting by separating epithelial cells from the surrounding stromal cells and managing them into a highly interconnected polarized cell monolayer [66]. In the salivary epithelium, tight junctions (TJs) and anchoring junctions such as adherens junctions and desmosomes control cell–cell interactions. TJs are located in the uppermost region of the lateral plasma membrane and include transmembrane proteins such as claudins, occludins, and anchoring protein ZO-1 [90, 91]. Adherens junctions provide the gland with mechanical support by connecting the actin cytoskeleton of neighboring epithelial cells. These junctions are located below tight TJs on the lateral plasma membrane and contain the transmembrane protein E-cadherin and anchoring proteins catenins, α-actinin, and vinculin. The intermembrane barrier created by TJs provides cell polarity. It prevents the lateral diffusion of membrane proteins between the apical and basolateral membrane domains and maintains the transepithelial ion gradients by prohibiting the free movement of ions through the paracellular space [90–93].

Cell polarization involves E-cadherin dimerization of two adjacent epithelial cells, followed by the subsequent enrollment of adherens junction, TJ, and cell polarity proteins, such as the partitioning defective (PAR) complex and the activation of Rho GTPases needed for downstream signaling [94, 95]. Cell polarity is regulated and controlled by the PAR complex, Crumbs complex, and scribble complex, irregularly localized to specific cell membrane areas.

BM components stimulate cell polarity by providing binding sites for cell surface receptor β1-integrin, leading to the activation of Rho GTPase RAC1 [96]. The four key components of the BM are laminin, type IV collagen, nidogen/entactin, and heparan sulfate proteoglycan (perlecan/HSPG2) [97]. BM proteins (i.e., laminin, collagen type IV, perlecan, and nidogen) present in Matrigel® are needed to establish acinar polarity of human SMG cells (attested by ZO-1, claudins1-5, and occluding expression), [98]. Similarly, hydrogels containing some BM components activate the expression of one or more TJ proteins [72, 99, 100]. Human stem/progenitor cells encapsulated in HA-based hydrogels containing a peptide from perlecan expressed ZO-1 and differentiated into acini [72, 101]. Further, HA-based hydrogels allowed acinar cells to express aquaporin 5 (AQP5) [72, 101], an apical acinar cell marker [47, 102, 103]. The limited cell polarity of mouse SMG cell clusters encapsulated into fibrin-based hydrogels was improved when laminin peptides were cross-linked to fibrin [25]. Synthetic hydrogels made from poly(ethylene glycol) (PEG) [27, 69, 104] or poly(lactic-co-glycolic acid) (PLGA) are other candidate materials for keeping cell polarity within the hydrogel [105]. When combined with MMP-cleavable sequences, these hydrogels promoted the localization of NKCC1, ZO-1, and AQP5 in mouse SMG cells [27]. Enzymatically degradable materials allow SG cells to undergo dynamic morphogenesis during their growth and proliferation and promote cell polarization by clearing a path in the hydrogel. Accordingly, in vitro models of ECM for SG tissue would provide practical tools to study the effect of different cues on salivary cells' polarity and help to understand the impact of various parameters on cell polarity.

### SG cells functionality

Saliva secretion is one of the primary functions of SGs and involves several cell types. As mentioned in previous sections, SGs mainly contain three different cell types: acinar cells, ductal cells, and myoepithelial cells. The acinar cells are specialized secretory cells that can be either serous or mucous [106]. TJ proteins seal acinar cells and form a barrier to the movement of large solutes between the apical/luminal and basal/stromal sides, ensuring no backflow of proteins secreted in saliva [107]. In response to nerve stimulation, the activation of Ca^2+^-regulated K^+^ and Cl^−^ channels induces an accumulation of Cl^−^ in the acinar lumen leading to subsequent Na^+^ movement from the interstitium into the acinar lumen to maintain electroneutrality. As a result of the formation of an osmotic gradient, water move to the apical lumen through the presence of AQP5. After that, saliva composition is modified via the reabsorption of sodium chloride and secretion of potassium bicarbonate in the ductal network. Final hypotonic saliva then enters the mouth [108, 109]. Acinar cells also secrete many proteins, including α-amylase (used as an acinar cell marker), salivary peroxidase, phosphatases, hydrolases, dehydrogenases, arginase, esterases, proline-rich, histatin-rich, and tyrosine-rich proteins playing a role in food breakdown and oral health maintenance [110, 111]. As acinar cells play a crucial role in saliva secretion, the repopulation and regeneration of SG acinar cells represent significant challenges for tissue engineering. Studies suggest that acinar cell markers are downregulated when acinar cells undergo cellular stress or injury [73]. Work is ongoing to identify cues and molecular pathways that encourage acinar cells to regenerate [112]. In native tissue, acinar cells tend to be repopulated through self-duplication [113], but ex vivo acinar cell proliferation and maintenance of differentiation have been challenging to achieve to date [14, 114]. The modification of substrate or hydrogels in which acinar cells are grown or encapsulated could provide some solutions to this problem. Understanding the role played by ECM in acinar cell proliferation, and differentiation allowed researchers to partially overcome this challenge [68, 115]. Results indicated that MMP-cleavable hydrogels are the most successful material in maintaining acinar cells phenotype, proliferation, and function [27]. However, acinar cells often lose their polarity and phenotype in long-term culture, which is indicated by a reduction in the expression of acinar cell markers such as MIST1 and AQP5 [104]. The expression of SOX2 is required to replace secretory cells in mouse models [116]. In addition, the proliferation of SOX2-positive cells could be promoted by acetylcholine, suggesting that acinar cell proliferation can be controlled by incorporating neural signals [112]. A detailed understanding of these interactions requires the development of biomimetic model hydrogel systems in which various cell types, including acinar cells, ductal cells, myoepithelial, and neuronal cells, can be co-cultured.

Many hydrogel formulations such as Matrigel^®^ [98, 117], hyaluronic acid (HA)-based hydrogels [13, 111], and enzymatically degradable PEG-based hydrogels [27] have been reported as suitable candidate materials to maintain α-amylase secretion in acinar cells. Functional responses of acinar cells can also be evaluated by measuring calcium oscillations occurring upon stimulation with β-adrenergic or muscarinic agonists [118, 119]. Such functional response was evaluated in acinar-like cells grown in HA gels [13].

To bioengineer a functional SG producing suitable saliva formulation, it may be necessary to integrate ductal cells as they are essential to change the salivary composition [11, 120] and may overcome protein accumulation within acini lumen that may hinder organoid systems. Engineering an acini-like structure interconnected with a ductal network to transport saliva and secreted proteins to an exterior port represent a major hurdle to overcome in the future.

### Innervation and vascularization

Parasympathetic and sympathetic innervation plays a role in the control of saliva secretion by inducing fluid-rich and protein-rich secretion [109]. Both β-adrenergic and muscarinic agonists can modulate the epithelial structures and function in engineered SG microstructure [13]. Further, neurotrophic factors such as Neurturin (NRTN) can control parasympathetic submandibular ganglion (PSG) function, promote neurite outgrowth and viability, and influence SG branching morphogenesis [121–123]. On the other hand, vascularization in SG requires integrated sympathetic, parasympathetic, and sensory input [118]. Accordingly, artificially engineered implants must provide the capacity to involve local innervation, either by direct secretion of neurotrophic growth factors or by supplementing 3D scaffolds with selected neurotrophins. Recently, transplantation of 3D culture of SG functional organoids and human dental pulp stem cells generated by magnetic 3D bioassembly promoted epithelial and neuronal growth in damaged irradiated mouse SG [22]. The combination of neuronal signaling cues, nanomaterials, and advanced microfabrication methods will open new avenues in SG tissue engineering.

SGs are also surrounded by a dense network of blood vessels that delivers oxygen to salivary cells, removes cellular waste, and provides the gland with an ample supply of fluid needed for saliva formation [118]. SGs are vascularized through the proliferation and migration of endothelial cells from preformed arteries [124]. Several soluble/paracrine strategies have been used to promote angiogenesis: release of vascular endothelial growth factor (VEGF) and platelet-derived growth factor-BB (PDGF-BB) from biodegradable PLGA scaffold [125], immobilization of ephrin A1 conjugated to PEG diacrylate hydrogels [126], covalent immobilization of PDGF-BB and FGF-2 to scaffolds [127–129]. Mesenchymal stem cells (MSCs) also improved tissue vascularization by forming spheroid aggregates that increased VEGF and FGF-2 [130, 131]. Layer-by-layer (LbL) cell coating method allowed the preparation of in vitro oral mucosa models in which the blood vessels were made from human umbilical vein endothelial cells [132]. However, the ability to construct large vasculature networks to develop more complex SGs requires proper ECM proteins [133]. Incorporating these vascular networks into existing or peptide-modified hydrogels might be beneficial for SG tissue engineering [133]. Furthermore, the nervous and vascular systems are two critical features in regulating outcomes of transferring approach for any artificial model in SG regeneration [134]. Despite advancing knowledge regarding the cues and mechanisms involved in SG innervation and vascularization, the development of innervated and vascularized SG remains an open challenge.

## Tissue engineering approach in salivary gland regeneration

**Fig. 3:**
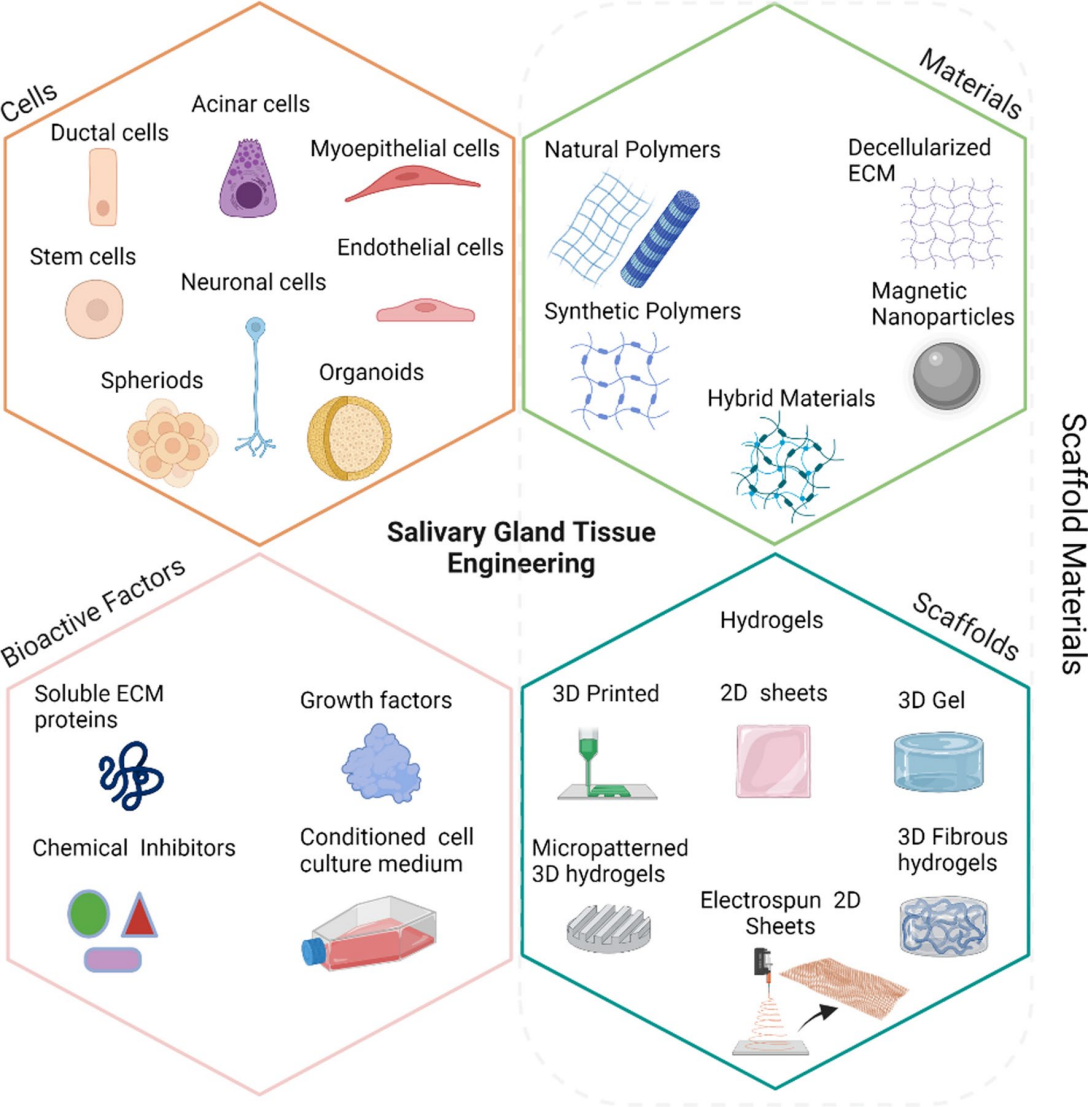
Schematic diagram of the essential cues for salivary glands tissue engineering (figure drawn using Biorender.com)

Cells, bioactive factors, and biomaterials are three critical cues that need to be used in an optimized combination to engineer whole organs and tissues. The combination of these different elements for preparing a SG bioengineered model is summarized in Fig. 3. All utilized cells, biochemical factors, and materials reported for SG tissue engineering applications are outlined below. Different scaffold design and fabrication strategies are discussed in "Strategies and fabrication methods in SG bioengineering".

### Cell sources

Cells represent the central aspects of SG regeneration via cell therapy approaches or the tissue engineering paradigm. Single cells [135] or cells retaining their 3D in vivo spatial organization can be obtained following SG dissociation [136]. Single cells can be sorted into specific parenchymal and stromal subpopulations using flow cytometry and/or selective enhancement during in vitro culture [53]. The challenge lies in assembling implantable and secretory tissue models by mixing stem/progenitor cells from adult SG with a biocompatible and biodegradable 3D scaffold. Also, establishing the efficacy of cell sheets or cell-seeded matrix in SG regeneration requires demonstration that the cells survive and are functional for a clinically-relevant duration, which still is a long-standing challenge in SG tissue bioengineering [137].

#### Salivary gland progenitor and adult stem cells

Defining and distinguishing the difference between progenitor and stem cells is critical since, despite the ambiguous definitions, they are not equivalent and show distinct properties. Progenitor cells can divide and differentiate only into specific types of cells for a limited number of times and cannot self-renew, while stem cells can replicate indefinitely, giving rise to both undifferentiated and differentiated progeny [138]. Both the embryonic and adult SG show multiple progenitor populations characterized by the expression of nuclear, cytoplasmic, and cell surface markers [122]. SG progenitor cells express the receptor tyrosine kinase c-Kit as a selective marker for regenerating damaged SMG in a mouse model [139]. A combination of four surface markers (Lin-CD24 + c-Kit + Sca1+) characterizes a subset of SMG progenitor cells that showed the highest spheroid-forming efficiency in culture and a robust multilineage regenerative ability when transplanted into irradiated mouse SMG [140]. The peripheral epithelial endbud cells express Keratin 14 (K14) in addition to c-Kit. The Kit + K14 + cells are involved in ductal morphogenesis. They establish a communication with the surrounding neuronal niche and proximal keratin 5 (K5) + epithelial progenitors thanks to the release of NRTN promoting parasympathetic nerve survival and axon extension that support the K5 + progenitors and sustains their ductal differentiation together with EGFR signaling [122]. Genetic lineage tracing has shown that K14^+^ cells are a multipotent epithelial progenitor population within SMG as they give rise to acinar, myoepithelial, and ductal cells, as well as K5 + -expressing cells [20, 122].

Sox-2 is an essential transcription factor for the maintenance of cell self-renewal and pluripotency and is involved in the formation of several tissues during development. Sox-2 + cells are stem/progenitor cells in the adult sublingual gland [116].

AcsI3 is another transcription factor expressed by adult progenitor cells. Genetic lineage tracing studies showed that AcsI3 + cells could generate a subset of the adult ductal and acinar cell descendants [122, 141]. While K14, K5, and c-Kit trace the lineage-restricted progenitors and can be expressed by different cell types within the ductal compartment, lineage-tracing studies have identified Acta2 (alpha-smooth muscle actin; also abbreviated as SMA) as myoepithelial cells marker proving the maintaining through self-duplication [142].

Different data are present in the scientific literature concerning the reproducibility of cell characteristics in engineered SG tissue. SG cells can form epithelial-like isolated clusters on plastic. On Matrigel-coated surfaces, SG can self-assemble into 3D acinar-like structures, expressing TJ proteins (i.e., occludin, claudin proteins, JAM-A, and ZO-1) as well as AQP5 [71, 143]. SG cell culture on microwell culture systems (hydrogel micropatterning and nanofibrous scaffold) led to acinar-like spheroids formation that showed a high expression of the acinar, ductal cell, and TJ markers, the ability to secrete α-amylase and response to adrenergic or cholinergic agonists (increase in intracellular calcium) [1, 144]. Furthermore, the myoepithelial cells had the functional ability to respond to neuronal signals, spread throughout collagen hydrogels and contract the surrounding hydrogel, and ensure the maintenance of spheroid organization to wrap around the spheroids [11, 145].

Despite slow SG cell turnover (> 60 days) and the lack of proof of the existence of a multipotent SG stem cell, studies have shown a regenerative potential of the gland [146]. Moreover, SG is characterized by a potential “absence” of a single and spatially segregated quiescent multipotent stem cell population, otherwise exhibiting the existence of multiple populations of proliferative stem/progenitor-like cells. In adult SG, MSC expressing surface antigens such as CD44, CD49f (integrin), CD90, and CD105 have been identified. SG stem cells can generate acinar, duct, and myoepithelial cell types. Potentially, stem cell populations can change their phenotype properties in response to the surrounding microenvironment exhibiting the plasticity to transition to intermediate, dedifferentiated, or alternated cell types [138]. MSC culture in Matrigel generates branched and aggregated structures resembling native SG acini and ducts [138, 147, 148].

#### Non salivary gland cells

Different cell therapy approaches involve using non-SG and/or non-epithelial cells to activate regenerative mechanisms in irradiated SGs. These reports include Bone Marrow (BM)-derived MSCs [149], BM-derived cells [150], human adipose-derived MSCs [151], SG-derived MSC-like cells [152], induced pluripotent stem cells (iPSC) [153], amniotic cells [154], embryonic stem cells (ESC) [155]. Among them, adipose-derived MSCs reduced cell apoptosis and tissue fibrosis, and both BM-MSC and SG-derived mesenchymal-like cells exerted immunosuppressive actions. In addition, BM-derived cells improved saliva secretion and microvessel density by inducing epithelial repair. Their positive effects may be mediated via paracrine pro-survival/proliferative actions on surrounding stem/progenitor cells. The paracrine effects of adipose and BM-derived cells are ensured by the released bioactive components (e.g., KGF, VEGF, IL6, and IGF1), also called “soup” [156–161], supporting anti-apoptotic and pro-proliferative actions. The presence of neurotrophic factors in the “soup” remains unclear. In addition, intravenous administration of “soup” may improve saliva production in rodent irradiated SG, whose clinical success directly depends on the remaining cells responding to paracrine action [162].

Pluripotent stem cells (PSCs), such as embryonic stem cells (ESCs) and induced pluripotent stem cells (iPSCs), have been used for SG regeneration. Successful SG regeneration has been achieved through orthotopic transplantation of a self-organized organ rudiment generated from PSCs in mice with defective parotids, in which some transcription factors (i.e., Sox9 and Foxc1) played some key roles in SG development [30]. Our finding that coculture of salivary glands cells with iPS cells formed differentiated salivary glands is significant, and future elucidation of the mechanism could lead to viable regeneration therapy of functional organs using iPS cells. Our study provides new insights for future research into the regeneration of organs, such as salivary glands.

iPS cells have also proved to be a promising treatment for SG cancer in vitro and in vivo in rats [163]. Co-culture of embryonic SG cells could form differentiated SG in vitro and in vivo in mouse SG, suggesting the iPS cell niche affects SG development and regeneration [153]. Furthermore, human SMG stem/progenitor cells (hSMGepiS/PCs)- formed SG organoids in vitro in response to FGF10 and generated an SG in vivo by responding to the mouse mesenchyme niche [164].

### Bioactive factors

**Fig. 4:**
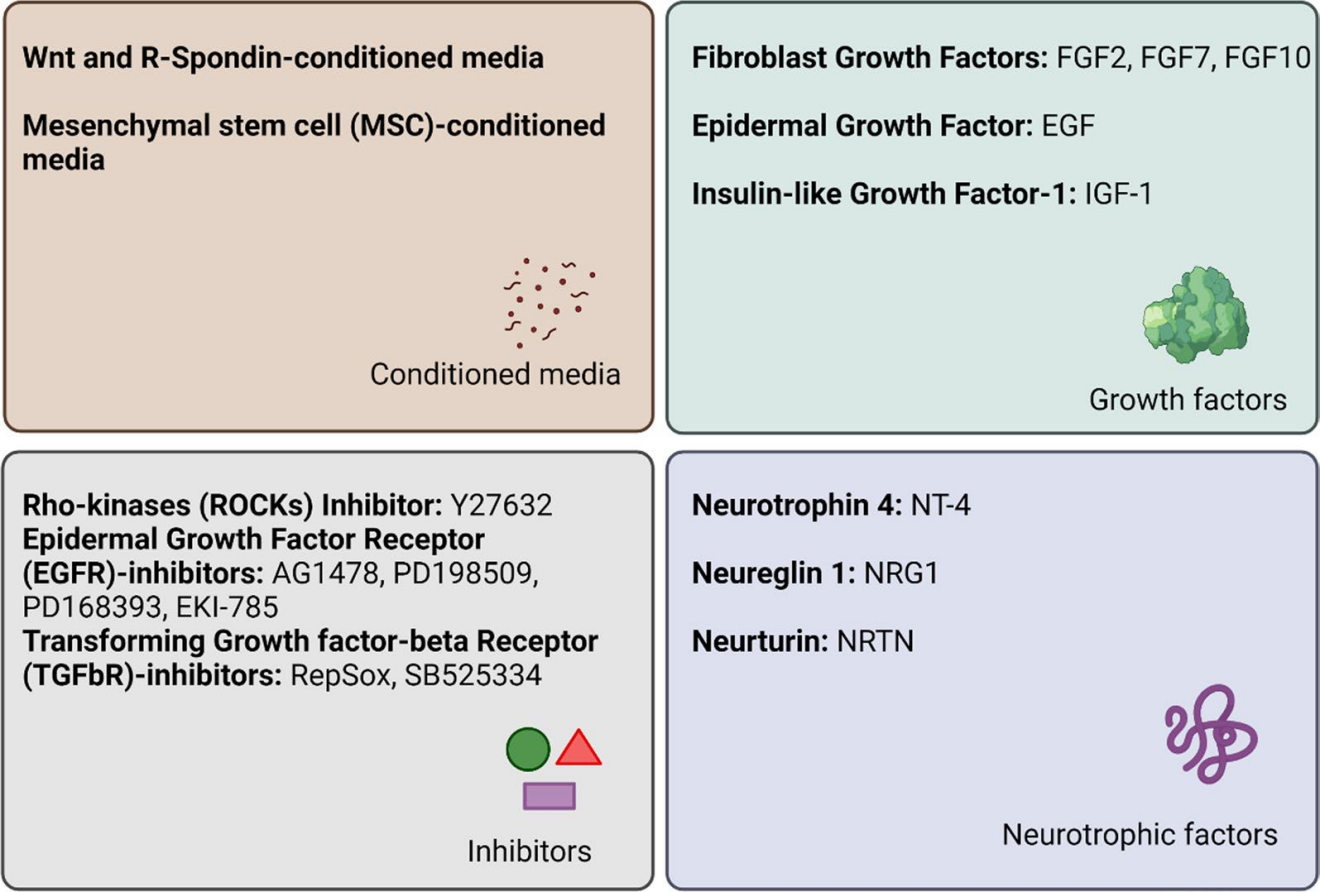
Soluble factors to improve cultured cell function in SG tissue engineering. (Figure drawn using Biorender.com)

Tissue-engineered scaffolds are intended to create and preserve differentiated cell phenotype, ensure basic functional units, and induce branching [71]. These functions rely on the use of specific bioactive factors added to the media or scaffolds. Cell culture media supplements include fetal bovine serum, glutamine, and antibiotics. In addition, conditioned media (containing released bioactive molecules) can increase the long-term expansion of salivary stem cells in vitro, showing increased population doubling and sphere-form efficiency. In fact, MSC-conditioned media induced an increment of acinar-like structures and AQP5 and K14 expression in the presence of laminin-111 [165]. As detailed above, soluble growth factors are fundamental in SG development, branching morphogenesis and end bud formation, cell proliferation, cell differentiation, initiation of innervation, and angiogenesis [166]. Chemical agents modulating specific signaling pathways can promote cell proliferation, motility, secretion, and proper cell shape. For example, ROCK inhibitors enhance cell growth, survival, proliferation, and α-amylase and Met protooncogene (c-Met) expression in SG culture [53, 167]. Moreover, soluble ECM proteins have primarily been used as supporting scaffolds to SG cells, promoting cell-ECM and cell–cell interactions. Fibronectin induced branching and ductal elongation. SG ECM extracts induced 3-D sphere-shaped structures and acinar markers, such as AQP5 and Muc-1 expression (53). Figure 4 summarizes the most common soluble cues for improving cultured cells’ morphogenesis, functionality, polarity, and promoting innervation in SG tissue engineering.

### Scaffolds materials

The nature of scaffold material plays a key role in mimicking natural tissues' mechanical, physiochemical, and biological features. Hence, in recent years, various scaffold materials have been investigated for SG tissue engineering, which can be sorted into three categories: naturally-derived biomaterials, synthetic polymers, and hybrid scaffolds materials.

#### Naturally derived biomaterials

Matrigel is one of the frequently used biomaterials for SG tissue engineering applications [98, 143], promoting cell attachment and differentiation in vitro [168, 169]. However, it is not an appropriate material for clinical translation due to its animal origin, and it is not easy to study the effect of its specific protein component on cell behavior. Therefore, using limited numbers of precisely defined BM components helps to identify a relationship between the activity of cells and a particular ECM constituent. For instance, Laminin, the major component of Matrigel, plays a critical role in SG development and morphogenesis [165], but the entire Laminin-1 sequence can cause unwanted side effects, such as tumorigenesis, degradation, and immune reactions, making it unsuitable for clinical applications [170]. Thus, peptides derived from ECM components are other natural components that can be incorporated into scaffolds to support cell–matrix attachment and cell–cell adhesion. Some laminin-derived peptides can promote human SG spheroids formation, branching morphogenesis, and SG functionality [25, 171]. Primary human salivary stem/progenitor cells grown on a scaffold containing a peptide from domain IV of perlecan/ heparan sulfate proteoglycan (HSPG2) formed acini-like spheroids [100, 172]. In addition, human-derived hydrogels based on fibronectin and placenta BM extracts stimulated morphological and functional differentiation of primary human SG epithelial cells to create polarized salivary acinar-like structures [173]. Also, fibronectin-derived RGD peptides and collagen I-derived MMP-sensitive (PQ) peptides are commonly used as cleavable crosslinkers that can be integrated into a synthetic polymer network to aid in thiol-ene polymerizations of PEG hydrogels [104].

The whole decellularized SG ECM can also be used as another naturally-based biomaterial. Indeed, repopulation of decellularized rat SMG with SG cells indicated significant cellular adhesion, differentiation, and gland-like tissue formation in vitro [174]. Decellularized ECM prepared from human SMG biopsies is also used as a substrate for seeding and growing human epithelial cells and fibroblasts [175]. However, biomaterials derived from animal or human tissues lack tunability and reproducibility and increase the risk of tumorigenic or immunogenic reactions [71].

Scaffolds can also be made up of biologically-derived polysaccharides and proteins [176]. HA is one of the popular hydrogel biomaterials in tissue engineering. Recently, HA-based hydrogels allowed a 3D culture of SG spheroids using primary salivary human stem/progenitor cells [26]. Moreover, arginine-glycine-aspartic acid-modified alginate-based hydrogels and chitin/chitosan-based scaffolds provided a suitable environment for enhancing SMG bud expansion and cleft formation as well as SG branch formation to produce essential ECM [177–181]. Collagen type I, fibrin, fibronectin, and silk fibroin proteins are other standard biomaterials that have been used for SG tissue engineering applications and reported to promote SG epithelial cell growth, aggregate formation, and differentiation as well as matrix formation [11, 71, 182] . Recently, a combination of egg components such as egg white and egg yolk plasma proved to be a biocompatible and cost-effective material for 3D SG mesenchymal and epithelial cell survival [183]. Nevertheless, for all natural polymers, due to their solubility in water and poor mechanical properties, some chemical modification and crosslinking strategies are necessary for their use as scaffold biomaterials [183].

#### Synthetic polymers

Compared with natural polymers, synthetic biomaterials provide adjustable physicochemical and mechanical properties, two crucial environmental cues for cell growth and differentiation [71], and can be produced in large amounts without any limitation of scalability and extraction process. Moreover, synthetic materials can provide a chemically defined, xenogenic-free environment that can be modified for desired outcomes and provide reproducible results, which can be an alternative to Matrigel and other natural source materials [184]. The most common synthetic polymers for SG tissue engineering applications are PLGA [185] and PEG [27, 104]. Most commercially available synthetic materials are produced by ring-opening polymerization. The use of methacrylate-based polymerization of PEG-based hydrogels induces a significant loss in SMG cell viability. In contrast, the use of thiol–ene polymerization of PEG-based hydrogels is more favorable to submandibular cell encapsulation [104]. Nevertheless, the encapsulated single cells in both hydrogels failed to form organized SG structures [104]. Encapsulation of pre-assembled multicellular spheroids in these types of hydrogels could enhance cell viability stimulate cell proliferation, as well as promote and preserve cell–cell contacts [104]. In addition, polyacrylamide gels, utilized to assess the effect of the substrate modulus on SMG regeneration, are physiologically compliant gels (0.48 kPa) as they allow higher SG branching morphogenesis than stiffer gels (20 kPa) [186]. Also, it was reported that PVDF compare to different synthetic biomaterials, including polyvinyl alcohol (PVA), ethylene vinyl alcohol (EVAL), polycarbonate (PC), promoted branching morphogenesis in serum-free in vitro culture [187, 188].

#### Hybrid materials

Hybrid scaffold biomaterials containing synthetic and natural polymers have been developed to improve the bioactivity of the polymer surface to enhance cell attachment and differentiation. When comparing the effects of several biodegradable polymer-based substrates (i.e., poly-l-lactic acid (PLLA), polyglycolic acid (PGA)) and their copolymers (PLGA) coated with different ECM proteins (i.e., collagen I, collagen IV, laminin, fibronectin, and gelatin), SG cells only attached to polymer disks with preabsorbed proteins and behaved similarly on PLLA and PGA [189]. Another study revealed that coating of PEG-terephthalate/poly (butylene terephthalate) scaffolds with Matrigel improved SG epithelial cell growth and morphology [190]. Also, it was reported that chitosan and laminin-coated PLGA nanofibers enhanced the proliferation of SG acinar and ductal cell lines [191].

## Strategies and fabrication methods in SG bioengineering

Generally, the bioengineering approach to restoring SG function can be divided into two main categories: cell-based techniques and cell-material-based strategies [192]. Recently, due to the importance of ECM and BM in SG structure and function, such as branching morphogenesis, polarity, and secretion, most recent studies have focused on using the second approach for SG tissue engineering purposes [71]. Indeed, biomaterials act as an ECM to prepare a suitable niche for cell attachment, proliferation, and differentiation [193]. Another classification in SG bioengineering is based on the in vitro cell culture dimensional model, which can be classified into two main groups. First, the 2D model when cells are seeded on the flat tissue culture plate surface (TCPS) or biocompatible polymers to create a polarized epithelial cell monolayer with the capability of unidirectional fluid secretion [71, 189]. Second, the 3D model, when cells form spheroid or are encapsulated and cultured in 3D matrices to proliferate, reconstitute the polarization and secretory acinar structures [13]. Studies related to these two model strategies are summarized in the following sections.

### 2D models

Several efforts have been made to develop suitable biomaterial scaffolds promoting the proliferation and differentiation of SG progenitor cells [71]. Porous membranes or scaffolds better support cell growth [194] and phenotype retention [182] than flat substrates. Human PG and SMG cells seeded onto Matrigel®-coated or uncoated TCPS promoted expressing α-amylase and AQP5 in 3D acinar-like units and forming ductal cell monolayers with TJs, respectively [98]. Primary human SG cells seeded on TCPS coated with perlecan domain IV peptide (PlnDIV) or Matrigel formed 3D acini-like salivary units expressing α-amylase [101]. Mimicking ECM and BM nanostructure showed that immortalized adult mouse or rat SG cell lines (SIMS, ductal; Par-C10, acinar) had more rounded and clustered morphology, as well as a reduced and more diffuse expression of focal adhesion proteins on fibrous scaffolds [185]. In addition, cell proliferation and polarization strongly depended on the surface coating of the nanofiber scaffolds [191]. To further mimic the architecture of the BM in SG epithelial cell niche, arrays of “craters” in polydimethylsiloxane (PDMS) lined with electrospun PLGA nanofibers were fabricated [195]. Increased crater curvature enhanced the average height of the SIMS cell monolayer, cell polarization, AQP5 expression (in both SIMS and Par-C10 cells), and TJ protein expression (occludin) in Par-C10 cells [195]. When human parotid epithelial cells were cultured on Matrigel, PEG, and micropatterned PEG hydrogel in the presence of an electrospun poly-caprolactone (PCL) nanofibrous microwells, the expression of salivary epithelial markers, TJ proteins, E-cadherin, and F-actin was increased in micropatterned hydrogel [196]. Recent studies related to the fabrication of fibrous and hydrogel scaffolds to evaluate some critical features in 2D salivary bioengineered models and related markers are summarized in Table 2.

**Table 2:** 2D scaffold-based strategies for SG tissue engineering

Material	Fabrication	Cell/tissue DC^a^, AC^b^	Evaluated marker/protein	Evaluated/obtained SG compartment					Refs.
				A	D	M	V	N	
PLGA	ELS	SIMS^a^	–		✓				[105]
PLGA/CH laminin-111	ELS, coating	SIMS^a^, SMG-C10^b^	AC marker: AQP5 CP markers: β-Actin, Occludin, ZO-1 CA marker: integrin-α_6_	✓	✓				[191]
PLGA	ELS	SIMS^a^, mSMG^a,b^ Par-C10^b^	CA markers: FAK, Paxillin, Talin, Vinculin	✓	✓				[185]
PLGA	ELS, Photolithography	SIMS^a^, Par-C10^b^	AC marker: AQP5 CP markers: E-cadherin, F-Actin, Occludin	✓	✓				[195]
PLGA/Elastin	ELS, covalent conjugation	SIMS^a^	CP markers: β-Actin, F-Actin, Occludin, ZO-1		✓				[197]
PLGA/PGS	core/shell ELS	SIMS^a^, NIH3T3	CP markers: F-Actin, ZO-1		✓				[198]
PVA/CH/PC	Mold Casting, Evaporation	mSMG^a,b^	ECM markers: Collagen type I, III			✓			[180]
AL/G4RGDS	Mold casting, Ionic CL	mSMG^a,b^	–		✓				[199]
PLGA	Mold casting	hPGAC^b^	AC markers: α-amylase, AQP5 CP markers: β-Actin, E-cadherin, ZO-1 CA markers: FAK, ILK, Snail, Vinculin	✓					[200]
Agarose/laminin peptides	Mold casting	hSG, SVEC4-10, hDFs	Cell attachment marker: FAK	✓			✓	✓	[201]
PA/Human plasma fibronectin	Photo CL	mSMG^a,b^	AC marker: AQP5 MC marker: SMA ECM marker: collagen IV	✓	✓	✓			[186]
PEG	Micropatterning, ELS	hPECs^a,b^	AC markers: α-amylase, AQP5 DC markers: KRT7, KRT18 CP markers: β-actin, E-cadherin, F-actin, Occludin, ZO-1	✓	✓				[196]
PEG	Photopatterning, ELS	hSGSCs	AC markers: α-amylase, AQP5 DC markers: KRT5, KRT7, KRT18, NHE1, SLC26C MC marker: KRT5 CP markers: E-cadherin, ZO-1 SC markers: CD90, HAS, ITGB1, KRT5, LGR5, NANOG, OCT4, POU5F1, SOX2, THY1	✓	✓				[144]
Fibrin/Laminin-111 peptides	Solidification	Par-C10^b^	CP marker: ZO-1	✓					[171]
Fibrin/Laminin-111 peptides	Solidification	Par-C10^b^	AC markers: AQP5, TMEM16A DC markers: KRT7 CP markers: F-actin, Na^+^/K^+^-ATPase, ZO-1	✓	✓				[202]
PC/Agarose/PCL/HA—catechol	Mold casting, Evaporation, 3D printing	mSMG^a,b^	AC marker: AQP5 MC marker: SMA EnC markers: CD31 SC marker: c-Kit, CD44	✓		✓	✓		[203]

*A* acinar, *AC* acinar cells, *AL* alginate, *CA* cell attachment, *CP* cell polarization, *CH* chitosan, *CL* crosslinking, *D* ductal, *DC* ductal cells, *EnC* endothelial cell, *ECM* extracellular matrix, *ELS* elctrospinning, *G4RGDS* Gly-Gly-Gly-Gly-Arg-Gly-Asp, *HA* hyaluronic acid, *hDFs* human dermal fibroblasts, *hPECs* human primary parotid epithelial cells, *hPG* human parotid gland, *hPGAC* human primary parotid gland acinar cells, *hSG* human submandibular gland ductal epithelial cell line, *hSGSC* human single clonal salivary gland stem cells, *KRT7* keratin 7, *KRT18* keratin 18, *M* myoepithelial, *MC* myoepithelial cell, *mSMG* ex vivo mouse submandibular gland cells, *N* neuronal, *NIH3T3* NIH 3T3 fibroblasts, *PA* polyacrylamide, *Par-C10* immortalized rat parotid gland acinar epithelial cell line, *PC* polycarbonate, *PCL* polycaprolactone, *PEG* poly(ethylene glycol), *PGS* poly glycerol sebacate, *PLGA* poly (lactic-*co*-glycolic acid), *PVA* polyvinyl alcohol, *SIMS* immortalized adult mouse submandibular salivary gland ductal epithelial cell line, *SMA* smooth muscle α-actin, *SMG-C10* immortalized rat submandibular gland acinar epithelial cell line, *SVEC4-10* immortalized mouse lymphoid endothelial cells, *SC* stem cell, *V* vascular

Although 2D cell models have been considered a reliable method for studying the in vitro characteristics of various cells, they cannot practically simulate the cell niche to provide appropriate cellular functions such as cell differentiation, proliferation, motility, and metabolism. Indeed, the 2D model cannot reproduce the in vivo interactions of cells or cells with the ECM.

### 3D models

Considering the inherent limitations of 2D cell models, 3D cell models have become predominant in mimicking physiological conditions and maintain cellular and tissue function by establishing proper cell signaling pathways and ECM interactions [204]. Various 3D culture methods can be used to form cell spheroids, such as spontaneous cell aggregation, hanging drop, magnetic 3D bioassembly, and rotating culture vessels [183, 205]. These 3D culture methods allow cell–cell interactions, cell polarity, and differentiation and recapitulate the ECM properties. Though, in these culture methods, there is no control in spheroid size which causes cell necrosis to occur in the core of the cell aggregates [1]. Following partial SG digestion, cells grown in a serum-free culture media form functional spheroids, with cells retaining their native ECM and expressing markers of cell polarization, acinar cell, and TJ [136]. However, after ten days, cell apoptosis started due to the uncontrollable increase in spheroid size, limitation in nutrients, and oxygen diffusion rate, an possible toxic effect of accumulated proteins within the core of the formed acini units [137]. But, culturing salivary spheroids in a 3D ECM-derived matrix can preserve their structural integrity over ten days [136, 175]. When organoids were produced by seeding cells into BM substrate like hydrogel matrix, they displayed better size uniformity, cell polarization, and cell–cell interactions than the abovementioned method. [206]. However, the main limitations of this method are the presence of xenogeneic components, uncontrollable degradation of the biomaterials, and a lack of mechanical stimulation [183, 207]. Therefore, human fibronectin and BM extracts, utilized as alternative hydrogel materials, promoted the differentiation of human SG cells into acinar-like structures [173, 208]. Further, recycled human tissue ECM (obtained by collecting the residual connective tissue that remained like a gelatinous mass) promoted the growth of epithelial cells exhibiting comparable morphology and proteins composition to the native SG tissue [175].

Recently, evaluating different physicochemical and mechanical effects of biomaterials on cells’ behavior opened a new 3D culture modality as a scaffold-based culture method [1]. In this method, the SG cells are seeded in the gel and form spheroids that can differentiate into acinar-like structures expressing TJ proteins (like occludin) and AQP5 [71, 98, 143]. Common hydrogel materials used in this method are tissue-extracted proteins like collagen, fibrin, and Matrigel [71]. Purified acinar cells seeded in a 2.5D HA-based hydrogel containing PlnDIV showed self-assembling into acini-like structures with cell junctions and a central lumen [72]. Similar results were reported with a 3D HA-based hydrogel culture system but fostered and maintained the growth and differentiation of functional, neurotransmitter-responsive acini-like spheroids for over 100 days [13]. Egg white-alginate blend 2.5D hydrogel is a cost-effective, and suitable material for SG tissue engineering applications as SG spheroid-like structure formation could be controlled by regulating alginate concentrations in the blend polymer solution [209].

Microwells of micropatterning and nanofibrous hydrogel scaffold have been used to promote more uniformed acinar-like spheroids [196]. Compared to 2D and 3D culture systems, the spheroids obtained with these microwell culture systems showed higher expression of acinar, ductal, and TJ markers as well as a significant amount of α-amylase secretion and intracellular calcium levels in response to adrenergic or cholinergic agonists [144]. It was shown that the formation of uniform spheroids needed a niche independent culture system within a serum-free culture medium [144, 196]. Recently, the 3D co-culture of NIH 3T3 fibroblasts and SIMS ductal SG epithelial cells in alginate microtubes via needle-to-needle microfluidic technique also represent a promising co-culture method for further understanding of epithelial and mesenchymal interaction during tissue morphogenesis and future practical applications in regenerative medicine [210].

Bioprinting technologies combined with other technology such as magnetic nanomaterials are alternative methods for adjusting the physical structure of the 3D culture model [211, 212]. There are two strategies for bioprinting via using magnetic forces. The first method is a label-free cell approach in which cells are suspended in paramagnetic liquid containing gadolinium (Gd3 +). The magnetized fluid is stimulated by applying a magnetic field and displacing cells toward regions with a low magnetic gradient [211]. This process can control the cell patterning and is a nozzle-free method that provides a rapid print of multicellular spheroids. But the major concern over this technology is the usage of cytotoxic paramagnetic suspending media and high concentrations of Gd3 + that could be toxic for tissue spheroids and increase the risk of imbalance osmotic pressure due to excessive use of ions in the paramagnetic medium [213]. The second magnetic-based bioprinting technology requires cell labeling with magnetic nanoparticles [6, 214]. In this method, the cells can be easily directed using mild magnetic forces, and spatial patterning of the 3D cell assembly into the desired morphological structure can be regulated by altering the magnetic field's shape or configuration. Besides, the size of the spheroids can be adjusted by tuning magnetic nanoparticle concentration, the number of cells, and magnet size [23, 214]. However, the requirement for specialized equipment and the risk of toxicity for cells limit its application [23]. Accordingly, another group proposed using gel egg yolk plasma (GEYP) as a more abundant, biocompatible, cost-effective material for tissue engineering applications. They showed that GEYP was successfully 3D printed with controlled geometrics [215]. However, they mentioned that bioprinting of SG cells is still in progress and needs more optimizations.

Recent studies related to the fabrication of a 3D scaffold-based model for salivary tissue engineering and evaluated markers are summarized in Table 3. Moreover, the pros and cons of 2D and 3D scaffold fabrication methods are listed in Table 4.

**Table 3:** 3D scaffold-based strategies for SG tissue engineering

Material	Fabrication	Cell/tissue DC^a^; AC^b^	Evaluated markers/proteins	Evaluated/obtained SG compartment					Refs.
				A	D	M	V	N	
HA, PlnDIV	Photo CL	hPECs^a,b^	AC marker: α-amylase CP marker: ZO-1	✓					[72]
HA	Thiol/acrylate CL	hPG AC	SC marker: CD44, CD168	✓					[216]
HA	Thiol/acrylate CL	hPG AC	AC marker: α-amylase, β1 adrenergic, β2 adrenergic, M3 muscarinic CP markers: β-catenin, Claudin-1, E-cadherin, ZO-1	✓					[13]
HA	Thiol/acrylate CL	hS/PCs	DC marker: KRT5 MC marker: KRT14 CP markers: β-catenin, F-actin, Occludin ECM markers: collagen IV, laminin SC markers: CD44, KRT5, KRT14	✓					[26]
HA, BM peptides	Thiol/acrylate CL	hS/PCs	AC markers: α-amylase, AQP5, HTN1, MIST1, MUC7, PIP, PSP, STATH DC markers: KRT5, KRT19, TFCP2L1 MC marker: KRT14 SC markers: c-Kit, ETV4, ETV5, KRT5, KRT14, MYC	✓	✓				[111]
HA, Peptides	Mold casting, photo CL	hS/PCs	AC markers: α-amylase DC marker: KRT5, KRT19 MC marker: KRT14, SMA CA markers: integrin α_1_, integrin α_5_, integrin β_1_, integrin β_4_ ECM markers: fibronectin; laminin SC markers: KRT5, KRT14	✓	✓	✓			[217]
HA, RGDSP	Thiol/acrylate CL	hS/PCs	AC markers: α-amylase, NKCC1 DC markers: KRT7, KRT19, TFCP2L1 CP markers: β-catenin; F-actin CA markers: CTGF, CYR61, GDF-15, SERPINE1, TGF-ß1, YAP, ECM markers: fibronectin, laminin SC markers: IGF2, KRT5, KRT14	✓	✓				[218]
Fibrin, GFRMG	Solidification, CL	mPG^a,b^, Par-C10^b^	AC marker: α-amylase CP markers: F-actin, ZO-1	✓					[117]
PEG, peptide	Thiol/acrylate CL	mSMG^a,b^	AC marker: Mist1, NKCC1 DC marker: KRT5 SC marker: KRT5	✓	✓				[104]
PEG, MMP-degradable peptide	Thiol-ene polymerization	mSMG^a,b^	AC markers: AQP5, IP3R3, MIST1, NKCC1, PIP DC marker: KRT5 CP marker: ZO-1 ECM markers: Collagen IV, Laminin SC marker: KRT5	✓	✓	✓			[27]
PEG, MMP-degradable peptide	Photo CL, Microbubble technology	mSMG^a,b^ hPEC^a,b^	AC markers: α-amylase, AQP5, Cst3, Cst10, IP3R3, Lyz2, MIST1, M3R, Muc5b, NKCC1, PIP, P2X_7_, P2Y_2_; Smr3a DC markers: KRT5, KRT7 MC marker: SMA CP markers: ZO-1 SC markers: KRT5	✓	✓				[219]
SF	Casting method lyophilization	rSGECs (SMG, PG)	AC marker: α-amylase ECM marker: collagen IV	✓		✓			[182]
PLL, Au^3+^/Fe^3+^ magnetic NPs	Magnetic 3D Bioprinting	hDPSC	AC markers: α-amylase, AQP5 DC marker: KRT5 MC marker: KRT14 SC markers: CD24, CD29, CD90, C-kit, KRT5, KRT14, SOX2 NC marker: ß-tubulin	✓	✓	✓		✓	[22]
dECM	Freeze drying	rSGSCs	AC markers: α-amylase DC marker: KRT5, KRT18 CP markers: Claudin-1, Claudin-3, E-cadherin, SC markers: CD44 c-Kit, c-Met, KRT5	✓	✓				[220]

*AC* acinar cells, *BM* basement membrane, *c-Met* met proto oncogene, *CA* cell attachment, *CP* cell polarization, *CL* crosslinking, *D* ductal, *DC* ductal cell, *dECM* decellularized extracellular matrix, *ECM* extracellular matrix, *GFRMG* growth factor reduced Matrigel, *HA* hyaluronic acid, *hDPSc* human dental pulp stem cell, *hPECs* human parotid epithelial cells, *hPG* human parotid gland, *hS/PCs* primary salivary human stem/progenitor cells, *mPG* mouse primary parotid gland cells, *M* myoepithelial, *MC* myoepithelial cell, *N* neuronal, *NC* neuronal cell, *NP* nanoparticles, *NS-SV-AC* immortalized acinar cell from human salivary gland, *Par-C10* immortalized rat parotid gland acinar epithelial cell line, *PG* parotid gland, *PLL* poly l-lysin, *RGDSP* integrin-binding peptide, *PlnDIV* peptide derived from domain IV of perlecan, *rSGECs* rat primary salivary gland epithelial cells, *rSGSCs* rat SG stem/progenitor cells, *SF* silk fibroin, *SG* salivary gland, *SC* stem cell, *SMG* submandibular salivary gland, *V* vascular

**Table 4:** Comparison of scaffold fabrication techniques in SG tissue engineering

Fabrication methods	Advantage	Challenge and disadvantage
Cast molding for hydrogel	Straightforward method, compatible with different materials, inexpensive, able to be scaled up	Lack of simulating micro and nanostructure of natural ECM
Electrospinning	Suitable to prepare a 2D sheet with nanostructure, able to be scaled up	limitation in choosing materials and solvents for fabrication, lack of 3D structure
Magnetic printing	Easy for organizing cells in unified order,	Toxic effect of nanomagnetic materials in high concentration for cells, high cost for preparing specific equipment, probability of imbalance osmotic pressure for cells. limitation to be scaled up, limitation in choosing materials for printing (viscosity, printability)
Bioprinting	Suitable to make a complex structure, suitable to provide a 3D environment for cells,	Limitations in using materials for printing (viscosity, printability, and crosslinking methods), Limitations to be scaled up, challenges related to control and characterizing cell polarity, self-organization, and functionality, difficulty in preparing a homogeneous mixture of cells and bioink
Micropatterning	Suitable to mimic the ECM microenvironment both in 2D and 3D scaffolds	Difficulties in designing very complicated microstructure, high cost, difficulty in scale-up
Microfluidic device	Easy to handle, compatible for single-cell study, controlling biochemical signals and characterizing cell functionality and behavior; compatible to be combined with other advanced technology such as 3D printing and nanotechnologies to mimic fluid flow in natural cell niche and simulate nano and microenvironment of natural tissue, compatible for different cells co-culture or multi-culture	Difficulties to design, high cost, difficulty in scale-up for complicated models, limitations in using materials due to their crosslinking method and fabrication process,

## Current challenges and future perspective

Despite the recent progress in SG tissue engineering, there are some challenges in bioengineered strategies related to the appropriate secretory function, vascularization, and innervation of the SG tissue models, as well as the risk of adverse host tissue response to the transferred biomaterials [33, 221]. In recent decades, advances in cellular and molecular biology opened a new vista to identify which type of cells, genes, and signaling pathways are playing critical roles in the morphogenesis and functionality of SG to develop new biomaterials and fabrication methods [222–225]. However, many questions related to understanding mechanotransduction mechanisms, branching patterns, and biochemical signaling during SG development still remain [226].

Cell-based strategies, especially using stem cells and cell-biomaterial-based techniques to prepare in vitro SG culture models, have revealed some new opportunities in SG bioengineering [1, 9, 71]. Nevertheless, it has not been clear how long the applied cells can maintain their viability and functionality after transferring them in vivo. It is also ambiguous how long it takes for some of these biomaterials to degrade in vivo and how they react in contact with blood flow, biochemical cues, and immune system, especially in patients with Sjögren’s Syndrome [11]. Although personalized medicine recently could help reduce immune cell penetration in implanted biomaterials, a multidisciplinary approach to engineer the desirable scaffold biomaterials for SG regeneration with optimal cell responses is still required. Moreover, one of the significant challenges in the translation of experimental therapies to clinical implementation is the selection of appropriate animal models for preclinical testing. In most cases, a large animal model is required to mimic as closely as possible the load and weight-bearing characteristics of the human body [227].

Further, despite the suitable polarization maintained by matrix mimetics, the secretory function of SG cells is limited [11]. Hence, incorporating combinatory approaches and increasing the complexity of models by introducing different cell types will offer proper SG cell functionality and secretion in artificial models. Few groups have currently tried to prepare a more complex SG model, but optimization of combined culture media and matrix in these models has not yet been achieved [53]. For example, the use of additive manufacturing methods such as bioprinting and microfluidic proposed that a combination of specific cell lines and growth factors could be a suitable method for achieving innervation and vascularization [6, 53, 207, 228, 229]. However, bioprinted tissue models are still restricted to millimeter size and constructed with only immature vascular networks that cannot support epithelial tissues' long-term culture and morphogenesis. Microfluidic chips are also complicated to fabricate and operate for cell culture and are not yet standardized [228, 229].

So, combining different additive manufacturing methods such as bioprinting and microfluidic with employing endothelial cells, neural cells, growth factors, and multifunctional biomaterials with suitable mechanical and biological properties to promote selective differentiation and organization of multiple cell types will be an objective for future studies [71]. It seems that tunable stimuli-responsive materials with dynamic mechanical properties may provide opportunities to manipulate the properties of engineered ECMs over appropriate time and length scales in future works [230–233]. Furthermore, DNA-cross-linked biomaterials with reversible control of matrix stiffness may offer an opportunity to understand the role of dynamic stiffness changes in epithelial morphogenesis [234]. Also, it seems that a combination of stem cell/gene therapy with 3D organotypic cell-based strategies will become the next generation of biomedical therapies to either restore a damaged SG or develop an in vitro SG model for transplantation in humans suffering from xerostomia [6, 235–237]. For instance, SG organoids derived from genetically modified induced pluripotent stem cells (iPSC) can be considered for fabricating SG models [2]. And all cell types present in the gland can be generated via programmed differentiation [115, 238, 239].

## Conclusion

Although significant progress in SG regenerative medicine, such as stem cell/gene therapy, has been reported over the past decade [41, 45, 240, 241], a conclusive approach to build a fully functional SG model based on cell-material-based strategies as a substitute for this exocrine organ remains a challenge [11, 242]. Future strategies will have to overcome the challenge of obtaining acinar-like structures surrounded by myoepithelial cells, interconnected in 3D with a network of ducts that will transport saliva to an exterior port, innervated and vascularized. Recently, different material and fabrication methods have been evaluated to prepare 2D and 3D SG models; however, the performance of these materials in vivo and the long-term functionality of the prepared models need to be studied further. Moreover, it remains unclear how long it will take for some of these materials to degrade and how stiff they need to stay intact long enough to support gland regeneration. Even though micro/nanofabrication techniques have provided an opportunity to simulate the cell microenvironment, the lack of fully understating the biomechanics and biochemistry of the natural SG tissue is one of the limitations to forming a complete practical SG model. Hence, developing a fully functional gland requires combined multidisciplinary efforts from various fields such as biology, genetics, biomaterials, engineering, and medicine to identify and mimic microenvironmental signals that are responsible for cell plasticity and functionality in SG tissue [240]. Translating ex vivo and in vitro findings obtained in rodents to human organoids derived from human progenitors remains a major hurdle due to the lower level of knowledge of the human SG morphogenesis and difficulties related to the availability of human cell sources. As SG development involves the interaction of various cells and neuronal signaling plays a vital role in its morphogenesis and functionality, innervation and vascularization, as well as co/multi-culture strategies in preparing 3D SG models, are anticipated to become promising approaches in SG tissue engineering.

## Data Availability

Not applicable.

## Abbreviations

A: Acinar
ABS: Acute bacterial sialadenitis
AC: Acinar cell
AL: Alginate
aPKC: Atypical protein kinase C
AQP5: Aquaporin 5
BM: Basement membrane
CA: Cell attachment
cAMP: Cyclic AMP
CBS: Chronic bacterial sialadenitis
Cdc42: Cell division control protein 42
CH: Chitosan
CL: Crosslinking
c-Met: Met proto oncogene
CP: Cell polarization
D: Ductal
DC: Ductal cell
dECM: Decellularized extracellular matrix
E: Epithelial
EnC: Endothelial cell
ECM: Extracellular matrix
EGF: Epidermal growth factor
EGFR: EGF Receptor
EHS: Engelbreth–Holm–Swarm
ELS: Electrospinning
ER: Endoplasmic reticulum
ERK-1/2: Mitogen-activated protein kinases
ESC: Embryonic stem cells
EVAL: Ethylene vinyl alcohol
FGF: Fibroblast growth factor
FGFR: FGF receptor
G4RGDS: Gly-Gly-Gly-Gly-Arg-Gly-Asp
GEYP: Gel egg yolk plasma
GFRMG: Growth factor reduced Matrigel
HA: Hyaluronic acid
hDFs: Human dermal fibroblasts
hDPSc: Human dental pulp stem cell
hPECs: Human parotid epithelial cells
hPG: Human parotid gland
hPGAC: Human primary parotid gland acinar cells
hS/PCs: Primary salivary human stem/progenitor cells
hSG: Human submandibular gland ductal epithelial cell line
hSGSC: Human single clonal salivary gland stem cells
HSPG: Heparan sulfate proteoglycan
iPSC: Induced pluripotent stem cells
KRT18: Keratin 18
KRT7: Keratin 7
LbL: Layer-by-layer
M: Myoepithelial
MC: Myoepithelial cell
MMP: Matrix metalloproteinase
mPG: Mouse primary parotid gland cells
MSCs: Mesenchymal stem cells
MSGs: Minor salivary glands
mSMG: Ex vivo mouse submandibular gland cells
N: Neuronal
NC: Neuronal cell
NIH3T3: NIH 3T3 fibroblasts
NP: Nanoparticles
NRTN: Neurturin
NS-SV-AC: Immortalized acinar cell from human salivary gland
PA: Polyacrylamide
PAR: Partitioning defective
Par-C10: Immortalized rat parotid gland acinar epithelial cell line
PATJ: PALS1-associated tight junction
PC: Polycarbonate
PCL: Polycaprolactone
PDGF: Platelet-derived growth factor
PDMS: Polydimethylsiloxane
PEG: Poly(ethylene glycol)
PG: Parotid gland
PGA: Polyglycolic acid
PGS: Poly glycerol sebacate
PI3K: Phosphatidyl-inositol-3-kinase
PLCγ1: Phospholipase Cγ1
PLGA: Poly(lactic-*co*-glycolic acid)
PLL: Poly l-lysin
PLLA: Poly-l-lactic acid
PlnDIV: Peptide derived from domain IV of perlecan
PNS: Parasympathetic nervous system
PSG: Parasympathetic submandibular ganglion
pSGECs: Primary salivary gland epithelial cells
PVA: Polyvinyl alcohol
PVDF: Polyvinylidene fluoride
RGDSP: Integrin-binding peptide
ROCK-1: Rho-associated coiled-coil-containing kinase 1
rSGECs: Rat primary salivary gland epithelial cells
rSGSCs: Rat SG stem/progenitor cells
SC: Stem cell
SF: Silk fibroin
SG: Salivary gland
SGSC: Human single clonal salivary gland stem cells
SIMS: Immortalized adult mouse submandibular salivary gland ductal epithelial cell line
SLG: Sublingual gland
SMA: Smooth muscle α-actin
SMG: Submandibular gland
SMG-C10: Immortalized rat submandibular gland acinar epithelial cell line
SNS: Sympathetic nervous system
SVEC4-10: Immortalized mouse lymphoid endothelial cells
TCPS: Tissue culture plate surface
TJs: Tight Junctions
V: Vascular
VEGF: Vascular endothelial growth factor
ZO-1: Zonula occludens-1
